# Remodeling of host membranes during herpesvirus assembly and egress

**DOI:** 10.1007/s13238-018-0577-9

**Published:** 2018-09-21

**Authors:** Ying Lv, Sheng Zhou, Shengyan Gao, Hongyu Deng

**Affiliations:** 1 CAS Key Laboratory of Infection and Immunity, Institute of Biophysics, Chinese Academy of Sciences, Beijing, 100101 China; 2 University of Chinese Academy of Sciences, Beijing, 100049 China; 3 CAS Center for Excellence in Biomacromolecules, Institute of Biophysics, Chinese Academy of Sciences, Beijing, 100101 China

**Keywords:** herpesviruses, assembly, egress, budding, fusion, membrane deformations

## Abstract

Many viruses, enveloped or non-enveloped, remodel host membrane structures for their replication, assembly and escape from host cells. Herpesviruses are important human pathogens and cause many diseases. As large enveloped DNA viruses, herpesviruses undergo several complex steps to complete their life cycles and produce infectious progenies. Firstly, herpesvirus assembly initiates in the nucleus, producing nucleocapsids that are too large to cross through the nuclear pores. Nascent nucleocapsids instead bud at the inner nuclear membrane to form primary enveloped virions in the perinuclear space followed by fusion of the primary envelopes with the outer nuclear membrane, to translocate the nucleocapsids into the cytoplasm. Secondly, nucleocapsids obtain a series of tegument proteins in the cytoplasm and bud into vesicles derived from host organelles to acquire viral envelopes. The vesicles are then transported to and fuse with the plasma membrane to release the mature virions to the extracellular space. Therefore, at least two budding and fusion events take place at cellular membrane structures during herpesviruses assembly and egress, which induce membrane deformations. In this review, we describe and discuss how herpesviruses exploit and remodel host membrane structures to assemble and escape from the host cell.

## Introduction

Viruses are intracellular parasites that hijack host cells to replicate themselves and produce infectious progenies. Following entry into host cells, viral genome uncoating, gene expression, genome replication, assembly and egress of new virions all take place at different intracellular compartments. Many viruses, including enveloped and non-enveloped viruses, exploit and remodel membrane structures to create distinctive spaces for these processes, and viral particles are transported between compartments in sequential order. For example, plus-stranded RNA viruses and some DNA viruses induce membrane structures derived from different cellular compartments to support the replication of their genomes in the cytoplasm. Among plus-stranded RNA viruses, RNA synthesis of hepaciviruses takes place in double-membrane structures, which are composed of two closely apposed membrane bilayers probably derived from the endoplasmic reticulum (ER). Togaviruses replicate in modified endosomal and lysosomal structures while nodaviruses remodel mitochondrial membranes for their replication (Miller and Krijnse-Locker, 2008). Herpesviruses, which are large, enveloped DNA viruses, replicate their genomes in the nucleus and remodel several host membranes for their assembly and egress.

**Figure 1:**
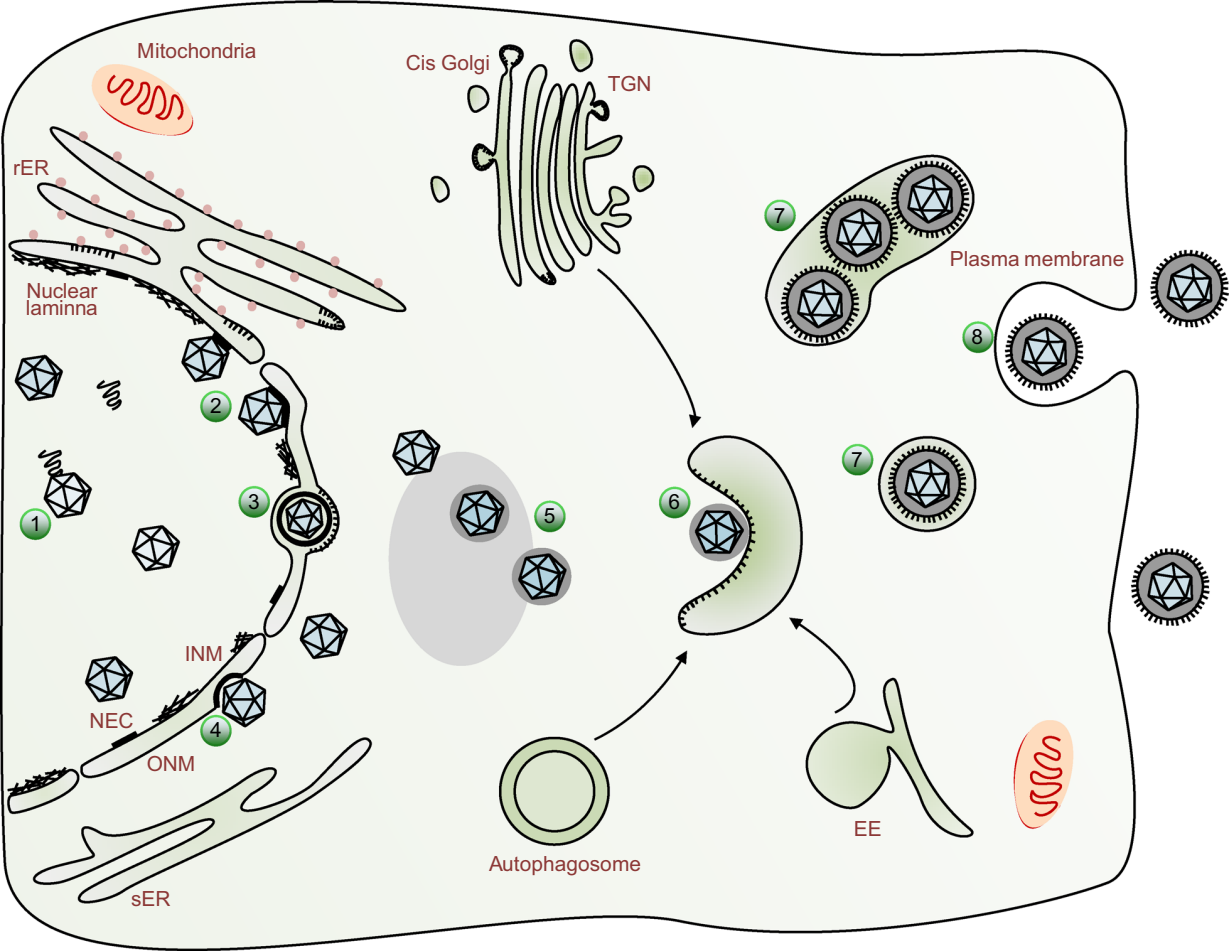
**A schematic representation of herpesvirus assembly and egress**. In the nucleus, newly synthesized viral genomes are packaged into the capsids (1). The nascent nucleocapsids traffic to the inner nuclear membrane (INM) where nuclear egress complex (NEC) are anchored and the nuclear lamina has been dissolved by host or viral kinases (2). Nucleocapsids bud at the INM, forming primary enveloped virions in the perinuclear space (3, primary envelopment). The primary envelopes of virions then fuse with the outer nuclear membrane (ONM), releasing the nucleocapsids into the cytoplasm (4, deenvelopment). In the cytoplasm, nucleocapsids acquire tegument proteins at electron-dense deposits, which is obvious in transmission electron microscopy micrograph (5). The tegumented capsids bud into special vesicles (6) that may derive from trans-Golgi network (TGN), early endosome (EE) or autophagosome, and form mature virions inside vesicles (7, secondary envelopment). Finally, these vesicles fuse with the plasma membrane and release mature virions to extracellular space (8)

Herpesviruses are classified into three subfamilies: alphaherpesviruses, betaherpesviruses and gammaherpesviruses (Davison et al., 2009). All herpesviruses share a common virion structure and similar proliferation strategies. An infectious virion consists of a 120–130 nm icosahedral capsid with a linear double-stranded DNA genome, a protein-containing tegument layer, and a host-membrane derived envelope spiked with virus-encoded glycoproteins. The life cycle of herpesviruses consists of several complex steps (Fig. 1). Upon initial binding to host cells, herpesviruses enter cells by fusion of the virion envelope with the plasma membrane (Spear and Longnecker, 2003). The capsids then interact with microtubules and are translocated to the nuclear envelope, where the linear viral genomic DNAs are injected into the nucleus through nuclear pores. After viral gene expression and viral DNA replication, the viral genomes are packaged into preformed capsids in the nucleus (Lee and Chen, 2010). The nascent nucleocapsids bud at the inner nuclear membrane to form primary virions in the perinuclear space, and then the envelopes of primary virions fuse with the outer nuclear membrane, releasing the nucleocapsids into the cytoplasm for further maturation. In the cytoplasm, nucleocapsids obtain 17–38 (or more) tegument proteins and bud into vesicles derived from host organelles. Finally, enveloped virions are released from the cells through fusion of the vesicles with the plasma membrane (Mettenleiter et al., 2009; Guo et al., 2010; Johnson and Baines, 2011).

Eukaryotic cells contain various membrane structures, which divide cells into different compartments to efficiently perform distinctive functions. As the interfaces of intracellular systems, membrane structures maintain the integrity of organelles and serve as carriers of intracellular trafficking to contact different organelles. Therefore the cellular membrane structures are dynamic and closely interrelated. Recent studies have attempted to address how cells regulate membrane systems to transport materials and how these systems are exploited by invading pathogens (Behnia and Munro, 2005). As described above, each step of herpesvirus life cycle is associated with intracellular structures, in particular cellular membranes and the cytoskeleton. There are at least two budding and fusion events that take place at cellular membrane structures during herpesvirus assembly and egress. In this review, we will describe how herpesviruses exploit and remodel host membrane structures to assemble and escape from the host cell. We will mainly focus on alphaherpesviruses, in particular the human pathogen herpes simplex virus type 1 (HSV-1) and the porcine pathogen pseudorabies virus (PrV), which have been extensively studied and are relatively better understood. Comparison with betaherpesviruses and gammaherpesviruses on certain aspects of virion assembly and egress will also be presented.

## Remodeling of the nuclear envelope for egress of nucleocapsids

### The architecture of the nuclear envelope

The nuclear envelope (NE) of mammalian cells is composed of inner nuclear membrane (INM), outer nuclear membrane (ONM), perinuclear space (PNS) and membrane-connected nuclear pore complexes (NPCs) (Stewart et al., 2007). PNS is a regular gap of 30 to 50 nm between INM and ONM, which is continuous with the ER luminal and maintained by the linker of nucleoskeleton and cytoskeleton (LINC) complex (Guttinger et al., 2009). Annular junctions of INM and ONM form nuclear pores occupied by NPCs. There are several thousand NPCs in the NE of a vertebrate cell, which serve as gates regulating the transport of macromolecules across the NE. Underlying the nucleoplasmic face of INM is the nuclear lamina. It consists of polymeric assembly of lamins (lamin A/C and B) and lamin-associated membrane proteins, providing structural support to the NE. In addition to serving as the interface between the nucleus and the cytoplasm in mammalian cells, the NE potentially acts as a physical barrier against virus infections.

### Conformational changes of the nuclear lamina induced by herpesvirus infection

After nucleocapsids are assembled in the nucleus, they cross through the NE to enter into the cytoplasm for subsequent maturation. However, the icosahedral herpesviral nucleocapsids are 120–130 nm in diameter, too large to traffic through the nuclear lamina network (with crossover spacing of ~15 nm) or the nuclear pores (the diameter of whose central channel is ~38 nm) (Alber et al., 2007; Goldberg et al., 2008; Lee and Chen, 2010). Therefore, nucleocapsids have to find ways to pass through the lamina network first in order to gain access to the INM. To achieve this, herpesviruses employ several strategies to modulate conformation of the lamina. Firstly, cellular and viral kinases are recruited to phosphorylate lamin A/C or lamin B during herpesvirus infection, resulting in disassembly of the lamina (Marschall et al., 2005; Park and Baines, 2006; Lee et al., 2008; Cano-Monreal et al., 2009; Sharma and Coen, 2014; Wang et al., 2014; Gershburg et al., 2015; Wu et al., 2016). This strategy is consistent with the well established notion that phosphorylation is responsible for the regulation of lamina assembly and disassembly during mitosis and apoptosis (Heald and McKeon, 1990; Peter et al., 1990; Kochin et al., 2014). Secondly, HSV-1 encoded nuclear egress complex (pUL34 and pUL31) binds to lamin A/C directly and disrupts lamin–lamin interactions (Reynolds et al., 2004). Thirdly, emerin and lamin B receptor, which are lamin-associated membrane proteins and serve as the connections between the lamin and the INM, are modified and redistributed during HSV-1 infection. HSV-1 encoded kinase pUS3 and cellular kinase PKCδ hyperphosphorylate emerin, leading to its relocalization and disrupting its connections with lamin proteins (Leach et al., 2007; Morris et al., 2007). Live-cell imaging and biochemical techniques also demonstrated a significant redistribution of lamin B receptor to the ER in HSV-1 infected cells (Scott and O’Hare, 2001). Fourthly, it has been shown that Ser22-specific lamin phosphorylation generates a binding motif for the cellular isomerase Pin1 in cells infected with human and animal alpha-, beta- and gamma-herpesviruses. Pin1 induces conformational change of lamin proteins, which facilitates disassembly of the nuclear lamina (Milbradt et al., 2010; Milbradt et al., 2016).

### Nuclear egress of herpesviral nucleocapsids

Once reaching the INM, the nucleocapsids cross through the NE to enter into the cytoplasm, a process designated as “nuclear egress”. Although alternative routes have been proposed for this process, the envelopment-deenvelopment model is largely accepted on the basis of ultrastructural observations and functional studies (Granzow et al., 2001; Peng et al., 2010). There are two steps according to this model. In the first step, called “primary envelopment”, nascent nucleocapsids bud at the INM, producing enveloped particles in the PNS, which could cause invaginations of the INM (Granzow et al., 2001; Peng et al., 2010; Reichelt et al., 2012; Villinger et al., 2015; Nanbo et al., 2018). In the second step, called “deenvelopment”, the envelope of viral particles fuses with the ONM and nucleocapsids are released into the cytoplasm. Nuclear expansion, which requires enlargement of nuclear membrane, is also observed during this process.

#### Primary envelopment: budding at the INM

The nuclear egress complex (NEC) of herpesviruses, composed of two conserved viral proteins, is essential for primary envelopment. In alphaherpesviruses such as HSV and PrV, the NEC consists of pUL34 and pUL31, whose orthologues include proteins encoded by the *UL50* and *UL53* genes in human cytomegalovirus (HCMV, a betaherpesvirus), the *BFRF1* and *BFLF2* genes in Epstein-Barr virus (EBV, a gamma-1 herpesvirus) and the *ORF67* and *ORF69* genes in Kaposi’s sarcoma-associated herpesvirus and murine gammaherpesvirus-68 (KSHV and MHV-68, gamma-2 herpesviruses). pUL34 is a type II integral membrane protein that localizes predominantly on the INM, ONM and ER by a transmembrane helix on its C-terminus (Shiba et al., 2000). pUL31 is a soluble phosphoprotein located in the nucleoplasm and is pulled to the surface of INM by its interaction with pUL34 (Chang and Roizman, 1993; Reynolds et al., 2001).

Budding of nucleocapsids is mediated by NEC at the INM during herpesviral infection. In the absence of NEC, primary envelopment is blocked and nucleocapsids accumulate in the nucleus (Klupp et al., 2000; Reynolds et al., 2001). Exogenous expression of PrV or KSHV NEC in mammalian or insect cells, respectively, results in the formation of vesicles in the PNS that resemble primary envelopes without nucleocapsids (Klupp et al., 2007; Desai et al., 2012). Expression of EBV BFRF1 induces vesicles derived from the NE, which are similar to those observed during EBV reactivation (Lee et al., 2012; Lee et al., 2016; Liu et al., 2018). Purified HSV-1 or PrV NEC drives membrane budding and scission in vitro in the absence of other proteins, indicating that the NEC functions as a minimal virus-encoded membrane-budding machinery during nuclear egress (Bigalke et al., 2014; Lorenz et al., 2015). The NEC coat architecture has been determined by combining X-ray scattering data of solubilized NEC structure and in situ cryoEM/T image of NEC arrays. The architecture reveals that the NEC forms an ordered lattice with two different hexameric layers. The unique structure and interaction between the two layers result in membrane curvature and scission (Hagen et al., 2015; Zeev-Ben-Mordehai et al., 2015).

Although the HSV-1 and PrV NEC are sufficient for vesiculation *in vitro*, they may not be sufficient in cells whose environment is more complex. NEC may recruit other factors to assist the primary envelopment process. For instance, UL47 and ICP22 of HSV-1 were found to colocalize with NEC at the INM in HSV-1-infected cells and promote primary envelopment, probably by interacting with proteins involved in viral nuclear egress and by regulating their functions (Liu et al., 2014; Maruzuru et al., 2014). UL21 and UL16 also play important roles on HSV-2 primary envelopment (Le Sage et al., 2013; Gao et al., 2017). In the absence of tegument protein ORF33 or ORF45, majority of MHV-68 capsids accumulate in the nucleus and their nuclear egress is significantly inhibited, indicating functional roles of ORF33 and ORF45 in this process (Guo et al., 2009; Jia et al., 2016). In addition to viral proteins, several host proteins involved in primary envelopment have been identified. For example, WDR5 was found to promote the primary envelopment of HCMV capsids, as knockdown of WDR5 expression induced substantially fewer infoldings of the INM and impaired the nuclear egress of HCMV capsids (Yang et al., 2018). During EBV infection, the cellular endosomal sorting complex required for transport (ESCRT) machinery participates in the scission of the INM. EBV BFRF1 protein recruits the ESCRT adaptor protein Alix to the nuclear rim of EBV-replicating cells. Inhibition of ESCRT machinery blocks the formation of BFRF1-induced vesicles and leads to the accumulation of viral genomes and capsid proteins in the nucleus (Lee et al., 2012). Furthermore, ubiquitination of BFRF1 is important for its induction of vesicle formation, which is probably mediated by itch, a ubiquitin ligase associated with BFRF1 (Lee et al., 2016).

#### Deenvelopment: fusion at the ONM

The molecular mechanism of deenvelopment is not well understood because of the difficulty to capture this process. Nonetheless, several viral and cellular proteins have been reported as regulators of HSV-1 deenvelopment. Similar to membrane fusion during virus entry, virion glycoproteins play important roles in mediating fusion between the envelope of the primary virions and the ONM. An HSV-1 mutant lacking both gB and gH fails to cross the nuclear envelope (Farnsworth et al., 2007). Phosphorylation of pUL31 and gB by the virally encoded kinase pUS3 has also proved important for the fusion process (Mou et al., 2009; Wisner et al., 2009). Cellular factors including p32, CD98 heavy chain (CD98hc) and β1 integrin are recruited to the nuclear membrane in HSV-1 infected cells (Hirohata et al., 2015; Liu et al., 2015). Inhibition of the expression or modification of any proteins mentioned above leads to aberrant accumulation of enveloped virions in the PNS or INM derived vesicles invaginating into the nucleoplasm (herniations). In cell lines constitutively expressing dominant negative forms of SUN1 and SUN2, which are components of the linker of nucleoskeleton and cytoskeleton (LINC) complex, primary enveloped virions of HSV-1 accumulate in the perinuclear space and escape into the ER, indicating that the intact LINC complex may promote the fusion of primary enveloped virions with the ONM (Klupp et al., 2017).

#### Nuclear expansion

Primary envelopment requires the INM for envelope formation, and deenvelopment results in fusion of the primary envelope with the ONM. Consequently, the INM would reduce constantly, while the ONM and its connected ER would enlarge. To our knowledge, however, such deformations have never been reported in cells infected with herpesviruses. Interestingly, nuclear expansion takes place at 8–10 h post infection (hpi) of HSV-1 (Simpson-Holley et al., 2005; Wild et al., 2009). Expansion of the nucleus demands enlargement of nuclear membrane to maintain membrane integrity, which requires a large amount of membrane composed of phospholipids. By measuring incorporation of [^3^H]-choline into the membrane, a recent study has confirmed that HSV-1 induces de novo synthesis of phospholipids and newly synthesized phospholipids are incorporated into nuclear and cytoplasmic membranes and viral envelopes (Sutter et al., 2012). In contrast to wild type HSV-1, mutant viruses lacking UL31 and UL34 fail to induce increases in the size of the nucleus, indicating that UL31 and UL34 are required for nuclear expansion (Simpson-Holley et al., 2005). The underlying mechanism warrants further investigation.

### Alternative routes for nuclear egress: NE breakdown and dilation of nuclear pores

Although nuclear egress is impaired in the absence of NEC, production of infectious virions is not completely abolished, suggesting the existence of alternative mechanism(s) for nuclear egress. On one hand, UL34- or UL31-negative mutants of PrV regain replication ability after serial passages in cell culture. Ultrastructural analyses have confirmed that these viral mutants escape from the nucleus via NE breakdown. Replication of the passaged mutants is impaired by inhibitors of cyclin-dependent kinase and MEK1/2, indicating involvement of mitosis-related processes in herpesvirus-induced NE breakdown (Grimm et al., 2012). HSV-1 infection also induces NE breakdown in Tor1a (a cellular ATPase) knock-out cells, while mutants lacking gB and gH inhibit NE breakdown. The dependence on gB and gH for NE breakdown suggest roles of membrane fusion proteins in this process (Maric et al., 2014).

On the other hand, gross morphological alterations of nuclear pores are observed in HSV-1 infected cells with several high-resolution imaging methods. HSV-1 infection results in decrease of nuclear pore numbers as well as dilation of nuclear pores, whose diameters are widened to more than 100 nm. Thus nucleocapsids may be able to pass through these enlarged nuclear pores to gain direct access to the cytoplasm (Hofemeister and O’Hare, 2008; Wild et al., 2009). However, whether herpesviruses exploit the nuclear pores for egress are still heavily debated and the molecular mechanisms resulting in the dilation of nuclear pores remain to be identified (Nagel et al., 2008).

## Enlargement and redistribution of the ER

The ER is an interconnected structure that spreads from the NE to the plasma membrane periphery. It is a multifunctional organelle, whose functions include synthesis and modification of proteins, synthesis of lipids, transport of membranes and proteins, and regulation of Ca^2+^ homeostasis (Baumann and Walz, 2001). Classically, the ER is divided into two domains, the smooth ER (sER) and the rough ER (rER) (Chen et al., 2013). Since the rER is connected with the ONM, the NE can also be considered as a part of the ER.

Morphometric analysis and calculation data have showed that the rER is temporarily enlarged at 12 hpi of HSV-1 and back to the normal size at 16 hpi. Since the number of capsids in the cytoplasm is lower at 12 hpi compared to 16 hpi, the temporary enlargement of rER may be related to the enhanced synthesis of lipids and proteins necessary for herpesvirus replication, rather than shifting of the enlarged ONM (Sutter et al., 2012). Immunofluorescence and electron microscopy analysis in a recent study has demonstrated that HSV-1 infection causes compression of the global ER architecture around the nuclear rim and UL34 is required for this alteration. It seems that HSV-1 induces remodeling of the ER for recruitment of regulators such as CD98hc, gB and gH to the nuclear membrane to facilitate nuclear egress of the viral particles (Maeda et al., 2017).

## Induction of vesicles for secondary envelopment

### Secondary envelopment

After translocation into the cytoplasm, the nucleocapsids obtain a series of tegument proteins and acquire their final envelopes via the secondary envelopment process, during which tegumented capsids bud into special vesicles to obtain membrane proteins and form mature virions inside the vesicles. Producing new virions at the site of secondary envelopment poses several requirements. For example, all viral glycoproteins need to be recruited to and stay at specific vesicles, and tegumented capsids need to be attached to these compartments (Peng et al., 2010). It is suggested that these vesicles are derived from host organelles such as trans-Golgi network (TGN) and early endosome (EE), based on research on HSV and PrV. In addition, some herpesviruses induce novel compartment or utilize autophagic membranes for their secondary envelopment.

### Compartments used for secondary envelopment

Studies on alphaherpesviruses have suggested that TGN and EE are utilized for secondary envelopment. HSV infection induces TGN reorganization from a tight juxtanuclear cluster to scattering dots throughout the entire cytoplasm (Campadelli et al., 1993; Sugimoto et al., 2008). Since a HSV virion contains capsid, tegument proteins and envelope proteins, accumulation of these structural proteins at one or a few specific sites seems to facilitate virus assembly (Johnson and Baines, 2011). By constructing a triply-fluorescent recombinant HSV that fuses different fluorescent protein individually with a glycoprotein, a tegument protein and a capsid protein, a study shows co-localization of HSV structural proteins with TGN marker (Sugimoto et al., 2008). This finding is in agreement with a study showing that extracellular HSV virions contain higher concentrations of sphingomyelin and phosphatidylserine, lipids typically enriched in the Golgi apparatus (van Genderen et al., 1994). In addition, HSV particles accumulate in organelles which co-fractionate with TGN and EE by membrane flotation assays (Harley et al., 2001). Furthermore, inhibiting the transport of cargos from TGN to plasma membrane causes accumulation of virions in TGN (Remillard-Labrosse et al., 2009). These lines of evidence are widely used to support the role of TGN in secondary envelopment of HSV and PrV. Recent data showed that, in addition to TGN, EE also contributes to HSV secondary envelopment since EE marker colocalizes with HSV structural proteins (Harley et al., 2001; Turcotte et al., 2005). However, during the late stage of herpesvirus infection, the membranes of TGN and EE undergo dramatic reorganization and the difference between TGN and EE blurs, therefore using markers to distinguish TGN and EE may not be accurate (Johnson and Baines, 2011).

Unlike alphaherpesviruses, the betaherpesvirus HCMV induces a novel compartment that contains protein markers including TGN46, Rab3 and ManII, indicating reorganization of TGN, EE and Golgi (Homman-Loudiyi et al., 2003). In addition, herpesvirus infection can induce autophagy, and autophagic membrane is also reported to be used by varicella-zoster virus (VZV, an alphaherpesvirus) and Epstein-Barr virus (EBV, a gammaherpesvirus) for their secondary envelopment (Granato et al., 2014; Nowag et al., 2014; Buckingham et al., 2016; Munz, 2017).

### Assembly of viral structural proteins at secondary envelopment sites

After tegumentation, capsids acquire their final envelopes at TGN, EE or membranes derived from them, where mature membrane glycoproteins are incorporated. These glycoproteins containing oligosaccharide chains covalently attached to polypeptide are guided to TGN by signal peptides (Gu et al., 2001). The sorting sequence of glycoproteins is important for their assembly into virions. For example, recruitment of gE of VZV to TGN depends on an AYRV motif and an acidic amino acid rich domain in the cytoplasm (Zhu et al., 1996). Similar to that of VZV, the cytoplasmic domain of gE of HSV is responsible for its accumulation in TGN in the early stage of infection (McMillan and Johnson, 2001). gB of HSV and PrV also have signal peptides in the cytoplasmic domain which guide them to the TGN (Beitia Ortiz de Zarate et al., 2004; Van Minnebruggen et al., 2004). Other glycoproteins without signal sequences may be transported to TGN through interaction with membrane proteins that contain these motifs. The finding that glycoproteins contain targeting signals is consistent with the evidence that TGN plays a critical role in viral secondary envelopment.

In a virion, the gap between capsid and viral envelope is bridged by tegument proteins. Recruiting capsids to the sites of secondary envelopment depends on complicated protein-protein interaction networks including capsid protein-capsid protein interactions, capsid protein-tegument protein interactions, tegument protein-tegument protein interactions and tegument protein-glycoprotein interactions (Owen et al., 2015). UL11 and UL16, conserved in alpha-, beta- and gamma-herpesviruses, were reported to coordinately bind to gE and this process contributes to virion assembly and egress (Han et al., 2012). A HSV mutant lacking gE-gI causes large aggregates of unenveloped capsids in the cytoplasm (Farnsworth et al., 2003). An MHV-68 mutant lacking ORF33, the homologue of HSV UL16, also demonstrates a similar phenotype (Guo et al., 2009). These lines of evidence demonstrate the critical roles of tegument proteins in facilitating budding of capsids into vesicles, but whether host molecules participating in this process remains to be investigated.

### Autophagic process is involved in secondary envelopment

Autophagy is a process that mediates the degradation of cytoplasmic materials, such as damaged organelles, protein aggregates and exogenous pathogens. It forms a double-membrane structure containing cellular proteins or organelles and eventually fuses with lysosome to degrade cargos (Mizushima et al., 2010). The execution of autophagy involves more than 20 conserved gene products, termed autophagy related gene (Atg) proteins, which are required for the formation of autophagosome. This process can be divided into two steps including nucleation and elongation. The ULK1/Atg1 kinase complex is activated by dephosphorylation and forms a large complex with Atg13, Atg101 and FIP200. Another complex contains Beclin-1, p150, Atg14L and the class III phosphatidylinositol 3-phosphate kinase (PI(3)K) Vps34. The activated ULK1 and Beclin-1 complexes are important for the nucleation step at the site of autophagy membrane formation. The Atg5, Atg12 and Atg16L1, which conjugate Atg8 to phosphatidylethanolamine (PE) on the surface of autophagosomes, is required for the elongation step. There are six Atg8 homologues in mammalian cells, named microtubule associated protein 1 light chain 3A (LC3A), LC3B, LC3C, Gamma-aminobutyric acid receptor-associated protein (GABARAP), GABARAPL1 and GABARAPL2. Lipidated LC3 contributes to the docking of autophagy cargos or adaptor proteins, such as SQSTM1/p62, via LC3-interacting region (LIR) (Richetta and Faure, 2013).

While autophagy acts as a host defense mechanism, certain herpesviruses utilize autophagic flux for its final envelopment. VZV infection induces the formation of autophagosomes. Pharmacological inhibition of autophagic membrane formation leads to decreased VZV glycoprotein biosynthesis and diminished viral titers. In particular, electron micrographs demonstrate that although no VZV particles are observed in autophagosomes, LC3-II is detected in highly purified VZV virions (Buckingham et al., 2015). Research also showed similarities between VZV gE and Atg9/Atg16L1 trafficking pathways (Buckingham et al., 2016). Thus, VZV seems to utilize LC3-conjugated membrane for its secondary envelopment and Atgs may mediate virus envelopment via regulating the transport of virus membrane associated proteins. For EBV, viral infection can cause the accumulation of autophagic membrane by blocking its fusion with lysosome (Granato et al., 2014). Similar to what is observed for VZV, inhibition of autophagic membrane generation by silencing Atgs decreases release of viral particles and leads to the cytoplasmic accumulation of viral DNA. Stimulating autophagic membrane formation by rapamycin enhances production of infectious virions. Furthermore, LC3 is found in EBV particles, suggesting that LC3-coupled membranes participate in secondary envelopment of EBV virions (Nowag et al., 2014; Munz, 2017). In addition, when the expression of Atgs is knocked down by siRNA, the replication of HSV-1 is inhibited, indicating important yet unknown function of Atgs (Mauthe et al., 2016).

## Release of mature virions by vesicle transport and membrane fusion

Secondary envelopment produces enveloped virions within intracellular vesicles. These vesicles travel to the plasma membrane via fast, directional transport on microtubules. Fusion between the transported vesicles and the plasma membrane releases viral particles into the extracellular space. In the late stage of PrV infection, Rab GTPase family proteins Rab6a, Rab8a and Rab11a are located on the virion-containing vesicles, which may promote intracellular transport by recruiting microtubule motors. Exocytosis of nascent virions frequently occurs near LL5β complexes, which anchor stabilized microtubules to the plasma membrane and provide an efficient pathway linking the site of secondary envelopment to the site of egress (Hogue et al., 2014). To bypass cortical actin and fuse with the plasma membrane, the conformation of myosin Va, a protein involved in secretory granule trafficking, is altered during HSV-1 infection to facilitate the transport of virion- or glycoprotein-carrying intracellular vesicles from TGN or endosome to the plasma membrane (Roberts and Baines, 2010).

There is also evidence that HSV can infect adjacent cells through cell-cell junctions. Particles are sorted in TGN so that enveloped virions are delivered to the lateral cell surfaces rather than to the apical surfaces in epithelial cells. The HSV gE-gI complex contributes to this sorting. Virions that arrive at cell-cell junctions are positioned in direct contact with adjacent cells, thereby promoting cell-cell spread of viruses (Johnson et al., 2001). Establishment of latency in ganglia by HSV and VZV depends upon the capacity to navigate in neuronal axons. To do this, viral particles tether themselves to dyneins and kinesins that move along microtubules from cell bodies to axon tips (anterograde transport). The HSV gE-gI complex and membrane protein US9 are able to initiate the process of anterograde axonal transport, ensuring that the viral particles are transported from the cytoplasm into the most proximal segments of axons by promoting both the envelopment and sorting of virus particles in the cytoplasm of neurons (DuRaine et al., 2017).

## Conclusions and perspectives

While cellular membrane structures can serve as physical barriers against herpesvirus infections, herpesviruses have managed over millions of years of evolution to complete their life cycles and proliferate by exploiting and remodeling host membranes. As described above, the morphology and subcellular organization of many membrane-bound organelles are altered during herpesvirus infections (Table 1). For example, the NE is enlarged or broken down. The ER is compressed around the nuclear rim, which may be required for recruitment of factors involved in nuclear egress. TGN markers are scattered over the entire cytoplasm rather than being localized in a tight juxtanuclear cluster, possibly for optimal secondary envelopment.

**Table 1:** Overview of the membrane modifications during herpesvirus assembly and egress

Intracellular structures	Modifications of membrane structures	Herpesviruses	References
Nuclear	Enlarged PNS as well as invaginations of the INM	EHV-1, PrV HSV-1, ILTV, HCMV, VZV, EBV, MHV68	Granzow et al., (2001), Peng et al., (2010), Reichelt et al., (2012), Villinger et al., (2015), Nanbo et al., (2018)
	Nuclear expansion at 8–10 hpi	HSV-1	Simpson-Holley et al., (2005), Wild et al., (2009)
	Nuclear membrane breakdown	PrV, HSV-1	Grimm et al., (2012), Maric et al., (2014)
	Decrease of nuclear pore numbers and dilation of nuclear pores	HSV-1	Hofemeister and O’Hare, (2008), Wild et al., (2009)
ER	Temporary enlargement at 12 hpi and back to the normal size at 16 hpi	HSV-1	Sutter et al., (2012)
	Compression around the nuclear rim	HSV-1	Maeda et al., (2017)
Golgi apparatus	Significant enlargements of Golgi membranes at 9, 12 and 16 hpi	HSV-1	Sutter et al., (2012)
	Fragmentation, numerous smaller structures dispersed throughout the cytoplasm	HSV-1	Campadelli et al., (1993)
	Redistribution of TGN membranes to form multiple cytoplasmic compartments	HSV-1	Sugimoto et al., (2008)
Other organelles	Reorganization of TGN, EE and Golgi to form novel vacuole compartment for secondary envelopment	HCMV	Homman-Loudiyi et al., (2003)

Abbreviations: Alphaherpesviruses: equine herpesvirus 1 (EHV-1), pseudorabies virus (PrV), herpes simplex virus type 1 (HSV-1), infectious laryngotracheitis virus (ILTV), varicella zoster virus (VZV), Betaherpesviruses: human cytomegalovirus (HCMV), Gammaherpesviruses: Epstein-Barr virus (EBV), murine gammaherpesvirus-68 (MHV-68)

However, the detailed mechanisms and biological significance of membrane deformation during herpesvirus infections remain largely unknown. In addition to NEC, only a few viral and host factors involved in nuclear egress are identified and their molecular mechanisms are not clear yet. Although exogenous expression of NEC results in the formation of vesicles in cells, empty vesicles in PNS are rarely observed in herpesvirus infected cells. Thus, primary envelopment is under precise regulation by inhibitory and triggering factors, which have not been identified. Proteins involved in the primary envelopment like NEC may also function in the deenvelopment. However, since lack of these proteins inhibits the primary envelopment process, it is technically difficult to study their roles in the subsequent deenvelopment process. Furthermore, the attempt to identify organelles by protein markers via immunofluorescence assays and by morphology via electron microscopy is confounded by the fact that protein markers are in a constant flux because of close communications among organelles, especially during the late stage of herpesvirus infection, when many organelles are significantly remodeled and protein markers are redistributed. So it is difficult to identify the source of vesicles induced in secondary envelopment. Along this line, it has been reported that secondary envelopment of HCMV takes place in a novel compartment containing protein markers of TGN, EE and Glogi, indicating complex mechanisms of the membrane organization (Homman-Loudiyi et al., 2003). Finally, little is known about factors involved in virus release.

Remodeling of host membranes during herpesvirus assembly and egress is complex and dynamic. Traditional studies mainly observe the morphology of membrane structures with immunofluorescent or electron microscope (EM), which suffers from low resolution or lack of temporal information in living cells. With the development of new technology and methodology, it becomes possible to tackle these problems and bridge knowledge gaps between cell biology and structural biology. For example, live correlative light-EM (CLEM) combines EM imaging with live-cell fluorescence imaging and allows for integration of spatiotemporal information from fluorescence imaging and high-resolution structural data from cryo-electron tomography (cryo-ET) (Kobayashi et al., 2016; Hampton et al., 2017). It can be used for identifying organelles involved in secondary envelopment and observing the morphology of cellular organelles at different stages of herpesvirus infections. In addition, super-resolution live cell imaging methods such as structured illumination microscopy (Kurokawa et al., 2013; Li et al., 2015) may reveal dynamic details of membrane trafficking and deformation process during herpesvirus infections and provide new insights into how herpesviruses remodel host membranes for assembly and egress.
